# Genomic sequencing is required for identification of tuberculosis transmission in Hawaii

**DOI:** 10.1186/s12879-018-3502-1

**Published:** 2018-12-03

**Authors:** Kent J. Koster, Angela Largen, Jeffrey T. Foster, Kevin P. Drees, Lishi Qian, Ed Desmond, Xuehua Wan, Shaobin Hou, James T. Douglas

**Affiliations:** 1 University of Hawaii at Manoa, Honolulu, HI USA; 2 Hawaii State Department of Health, Honolulu, HI USA; 3 University of New Hampshire, Durham, NH USA; 4 California Department of Public Health, Richmond, CA USA; 5 Advanced Studies in Genomics, Proteomics and Bioinformatics, Honolulu, HI USA; 6 Present Address: Pathogen and Microbiome Institute, Northern Arizona University, Flagstaff, AZ USA

**Keywords:** *Mycobacterium tuberculosis*, Hawaii, Spoligotyping, Genomic epidemiology

## Abstract

**Background:**

Tuberculosis (TB) caused an estimated 1.4 million deaths and 10.4 million new cases globally in 2015. TB rates in the United States continue to steadily decline, yet rates in the State of Hawaii are perennially among the highest in the nation due to a continuous influx of immigrants from the Western Pacific and Asia. TB in Hawaii is composed of a unique distribution of genetic lineages, with the Beijing and Manila families of *Mycobacterium tuberculosis* (*Mtb*) comprising over two-thirds of TB cases. Standard fingerprinting methods (spoligotyping plus 24-loci Mycobacterial Interspersed Repetitive Units-Variable Number Tandem Repeats [MIRU-VNTR] fingerprinting) perform poorly when used to identify actual transmission clusters composed of isolates from these two families. Those typing methods typically group isolates from these families into large clusters of non-linked isolates with identical fingerprints. Next-generation whole-genome sequencing (WGS) provides a new tool for molecular epidemiology that can resolve clusters of isolates with identical spoligotyping and MIRU-VNTR fingerprints.

**Methods:**

We performed WGS and SNP analysis and evaluated epidemiological data to investigate 19 apparent TB transmission clusters in Hawaii from 2003 to 2017 in order to assess WGS’ ability to resolve putative *Mtb* clusters from the Beijing and Manila families. This project additionally investigated MIRU-VNTR allele prevalence to determine why standard *Mtb* fingerprinting fails to usefully distinguish actual transmission clusters from these two *Mtb* families.

**Results:**

WGS excluded transmission events in seven of these putative clusters, confirmed transmission in eight, and identified both transmission-linked and non-linked isolates in four. For epidemiologically identified clusters, while the sensitivity of MIRU-VNTR fingerprinting for identifying actual transmission clusters was found to be 100%, its specificity was only 28.6% relative to WGS. We identified that the Beijing and Manila families’ significantly lower Shannon evenness of MIRU-VNTR allele distributions than lineage 4 was the cause of standard fingerprinting’s poor performance when identifying transmission in Beijing and Manila family clusters.

**Conclusions:**

This study demonstrated that WGS is necessary for epidemiological investigation of TB in Hawaii and the Pacific.

**Electronic supplementary material:**

The online version of this article (10.1186/s12879-018-3502-1) contains supplementary material, which is available to authorized users.

## Background

The World Health Organization has shown that the worldwide tuberculosis (TB) epidemic is larger than was previously estimated [1]. Tuberculosis caused an estimated 1.4 million deaths in 2015, with an estimated 10.4 million new cases. TB rates in the United States continue to steadily drop, yet TB rates in the State of Hawaii remain steady [2]. Hawaii experienced an average of 120 incident cases per year from 2006 to 2017, ranging from a low of 114 in 2006 to a high of 136 in 2014. Hawaii currently displays the highest incidence rate of TB in the US, at 8.1 per 100,000 in 2017. Comparing this rate to the median US state rate of 1.8 per 100,000 illustrates the public health burden of TB in Hawaii. Of the 119 incident TB cases in Hawaii in 2016, 100 (84%) were non-US born, well above the national average of 68.5%. Furthermore, of those 100 cases, 69 were in persons born in the Philippines.

Thus, it is not surprising that Hawaii perennially experiences among the highest rates of TB cases in the United States due to a continuous influx of immigrants from the Western Pacific and Asian regions. As a result of this immigration pattern, TB in Hawaii is composed of a unique distribution of genetic lineages relative to the continental United States or Europe, but similar to the United States Affiliated Pacific Islands [3–5]. The Beijing and Manila families of *Mycobacterium tuberculosis* (*Mtb*) comprise over two-thirds of the TB cases in Hawaii [6, 7]. These families are defined by spoligotyping (reverse-line hybridization of 43 sequences complementary to CRISPR spacers), mycobacterial interspersed repetitive units–variable number of tandem repeats (MIRU-VNTR) patterns, and whole-genome single nucleotide polymorphism (SNP) phylogenies [6–10]. The Manila family has been shown to comprise the majority of *Mtb* lineage 1 and has spread into the Pacific islands with Filipino migration, while the Beijing family comprises the majority of lineage 2 and is the dominant family in East Asia [10]. In contrast, lineage 4, whose members are the most commonly found among TB cases in Europe and North America, contains a larger set of spoligotyping clades [4].

Potentially long latency periods in tuberculosis cases make molecular epidemiological tools an essential part of its control. IS*6110* restriction fragment length polymorphism (RFLP) typing historically represented the “gold standard” for *Mtb* genotyping [11]. However, IS*6110* typing is time consuming and labor intensive, and provides limited resolving power for clusters composed of isolates with low IS*6110* copy numbers [12, 13]. Two other methods, spoligotyping and MIRU-VNTR fingerprinting, are currently the standard employed by the Centers for Disease Control and Prevention (CDC) in the United States [8, 12, 14]. However, these fingerprinting methods still perform poorly when used to identify actual transmission. One study conducted in the English Midlands found that the positive predictive value (PPV) that two isolates with identical MIRU-VNTR fingerprints represent actual recent transmission between those cases was only 18.6% [15]. Furthermore, they found that this PPV varied by lineage, with lineage 4 displaying a PPV of 30.6%, while lineage 1 displayed a PPV of only 8.0% and lineage 2 only 13.8%. Even more previous work has demonstrated that these genetic fingerprinting methods perform poorly for identifying actual transmission of Beijing family isolates, showing that MIRU-VNTR fingerprinting is superior to IS*6110* when only lineage 4 isolates are being typed, but performs poorly when Beijing family isolates are being typed [16]. Multiple other studies have also indicated that 12-loci MIRU-VNTR is insufficient to resolve suspected Beijing family clusters, and that 24-loci MIRU-VNTR is similarly ineffective when Beijing family isolates are present [17–19]. However, similar studies are not available for the Manila family.

Attempts to optimize VNTR typing for the Beijing family have been proposed and implemented with the switch from 12-loci to 24-loci typing, but as we further demonstrate in this study, have failed to result in a comprehensively effective solution [12, 20]. The need for effective epidemiological tracking for the Beijing family is highlighted by this family’s association with drug resistance. The population structure of TB in areas of high drug resistance has been shown to be rapidly shifting towards the Beijing family, which specifically has a significantly higher rate of developing rifampin resistance [21]. Alarmingly, the Beijing family has been shown to manifest increased transmission fitness relative to a non-Beijing lineage while streptomycin resistant [22]. However, limited research has been performed on the Manila family, despite its dominance in Hawaii and the Philippines, and despite the prediction that rates of multiple drug resistant (MDR) TB in the Philippines will continue to increase [23].

As a result of the predominance of these two *Mtb* families in Hawaii and the Pacific, identifying autochthonous *Mtb* transmission is especially difficult both in Hawaii and throughout the Western Pacific Region. Although extensive TB screening is implemented in Hawaii (including requiring tuberculin skin tests prior to enrolment in education or prior to employment as a food-handler), frequent travel of Hawaii residents to visit family in high-incidence areas throughout the Pacific, combined with insufficient existing molecular fingerprinting methods, prevents TB controllers in Hawaii from developing a comprehensive understanding of local TB transmission. In this study, we examined the ability of CDC-standard genetic fingerprinting (spoligotyping plus 24-loci MIRU-VNTR fingerprinting) for *Mtb* to identify Beijing and Manila family transmission clusters, and attempted to identify the cause of its reduction in genotyping resolution compared to when applied to lineage 4. We previously observed that the Beijing and Manila families demonstrated lower allelic Shannon evenness at most MIRU-VNTR loci [J.T. Douglas unpublished data]. The Shannon diversity index is a measurement of diversity in a community that considers both the richness (total number of alleles at each MIRU-VNTR locus, in our case) present in the community and evenness (relative abundance) of each of those alleles. Our study utilizes this measurement to determine if certain *Mtb* genetic lineages possess a dominance of specific alleles (indicated by reduced Shannon evenness values) at any MIRU-VNTR loci that may explain why MIRU-VNTR performs poorly when utilized for molecular epidemiology on these lineages. Here, we utilized a dataset of all fully fingerprinted *Mtb* isolates recorded in Hawaii from 2002 through 2016 to further investigate this apparent cause for MIRU-VNTR’s poor ability to resolve apparent lineage 1 and 2 clusters relative to its considerably greater ability for lineage 4 clusters.

Our previous cooperation with the State of Hawaii Department of Health Tuberculosis Control Branch revealed that the CDC’s standard *Mtb* fingerprinting methodology was of limited epidemiological use for Hawaii’s TB clinicians. Large numbers of epidemiologically unrelated Beijing and Manila family isolates frequently shared identical fingerprints, and nearly all suspected transmission clusters also fingerprinted identically within those suspected clusters, preventing fingerprinting results from being a useful tool for confirming or disproving suspected transmission events.

Whole genome sequencing (WGS) has been shown to be able to identify specific transmission chains within fingerprinting clusters [24]. Advances in next-generation sequencing have resulted in the cost of WGS decreasing to the point where it is feasible for many laboratories to sequence most or all clustered isolates [25]. WGS is increasingly being employed for tuberculosis epidemiology, including identifying the transmission chains of a TB outbreak in British Columbia, Canada, verifying contact investigation-based links in an outbreak in San Francisco, California, and use in a large, retrospective observational study in the UK Midlands [26–28]. For this study, we selected 19 apparent TB transmission clusters that were identified by fingerprinting or epidemiological data in Hawaii from 2003 to 2017 and conducted Illumina whole genome sequencing to determine if WGS could be used to further resolve these clusters and identify the transmission connections among isolates.

Making full use of the resulting WGS dataset, we further examined isolates from clusters that WGS identified to represent actual transmission events and investigated which genes or regions were developing mutations that differentiated individual isolates in a cluster. Our previous work has identified virulence factor mutations in the Beijing and Manila families that may be involved in virulence or latency, and this work seeks to help us further characterize these historically under-studied families [29, 30].

## Methods

### Identification of clusters for WGS

Records of all genotyped tuberculosis cases processed by the Hawaii State Department of Health Tuberculosis Control Program from 2004 to 2016—as well as partial data from 2002, 2003, and 2017—were analyzed to identify fingerprinting clusters that possibly represented actual transmission clusters. One thousand sixty-one isolate records were available for analysis. Names were assigned to spoligotypes using the SpolDB4 database [31]. Genetic fingerprints, dates and locations, patient histories, and nursing contact investigation records were all considered in the selection of these clusters. Four large historic *Mtb* fingerprinting clusters in Hawaii were selected for investigation (Table 1).

**Table 1:** Sequenced *Mtb* Fingerprinting or Epidemiological Clusters

Cluster Name	Cluster Family	# of Isolates	# of WGS Isolates	# of SNP Loci in Cluster	SNP Range	Transmission Linked?
Large Clusters Identified by Identical Genetic Fingerprints						
Manila Cluster 1	Manila	23	3	178	73–148	No
Manila Cluster 2	Manila	24	2	161	–	No
Beijing Cluster 1	Beijing	11	4	63	0–52	Partial
Beijing Cluster 2	Beijing	7	7	0	–	Yes
Clusters Identified by Shared Uncommon Spoligotypes						
Manila-like Cluster 1	Manila	2	2	3	–	Yes
Manila-like Cluster 2	Manila	2	2	4	–	Yes
Beijing Cluster 5	Beijing	2	2	1	–	Yes
Manila-like Cluster 3	Manila-like	3	3	3	1–3	Yes
H3 Cluster 1	H3	3 (1)	3	1230	3–1230	Partial
Epidemiologically Identified Putative Clusters						
Manila Cluster 3	Manila	2	2	90	–	No
Manila Cluster 4	Manila	2	2	0	–	Yes
Manila Cluster 5	Manila	2	2	192	–	No
Manila Cluster 6	Manila	2	2	229	–	No
Beijing Cluster 3	Beijing	2	2	3	–	Yes
Mixed Cluster 2	U/Beijing	2	2	1153	–	No
U Cluster 1	U	4	4	131	1–117	Partial
Mixed Cluster 1	Beijing/Manila	3	3	1762	1–1762	Partial
Manila Cluster 7	Manila	2	2	142	–	No
Beijing Cluster 4	Beijing	2	2	0	–	Yes

# of WGS Isolates is the number of isolates from each cluster that were sequenced with Illumina whole genome sequencing. # of SNP Loci in Cluster is the total number of SNP loci possessing alleles that differentiate isolates within the cluster. SNP Range lists the smallest and largest numbers of SNPs between any two isolates in the cluster. Transmission Linked is marked as “No” if all isolates in the cluster differ by 12 SNPs or more, “Yes” if they differ by 5 SNPs or fewer, or “Partial” if some, but not all, isolates within the cluster differ by 5 SNPs or fewer. H3 Cluster 1 includes a fourth isolate that was not available for sequencing. Clusters are listed in the order in which they are discussed in the text

As we hypothesized that these large fingerprinting clusters did not represent actual transmission clusters due to their relatively high number of cases, geographic distribution throughout the state, and chronological diversity, we further selected five clusters with spoligotypes that were less common in Hawaii, including two clusters with “Manila-like” patterns, one cluster with an uncommon Beijing family pattern (000000000003751 versus the common 000000000003771), one cluster with no spoligotype match in SpolDB4, and one H3 cluster (which is common globally, but uncommon in Hawaii) in order to analyze clusters with greater suspected likelihood of being transmission-derived. Nineteen isolates were selected for WGS from those clusters in order to maximize chronological diversity for the largest clusters and to fully sequence the smaller clusters.

We further worked with staff at the State of Hawaii Tuberculosis Control Program’s Lanakila Tuberculosis Clinic, including doctors, nurses, and Tuberculosis Epidemiological Studies Consortium (TBESC) staff, to identify 17 epidemiologically-derived possible transmission clusters, of which ten had two or more isolates sent to CDC-contracted laboratories for genetic fingerprinting (Table 1). Twenty-one isolates from these clusters were selected for sequencing.

### Recall of state of Hawaii *Mtb* isolates

Twenty isolates were requested from the Michigan Department of Community Health, where they had been previously sent by the State of Hawaii for contracted fingerprinting, and where they had been archived. We received extracted DNA from those isolates. Sixty-one isolates were sent from the California Department of Public Health State Laboratory as “double-killed” sample preps using a treatment of immersion in 70% ethanol followed by heating at 80 °C for 1 h.

### DNA extraction and whole genome sequencing

DNA extraction was performed as previously described by the National Institute of Public Health and Environmental Protection (RIVM), Bilthoven, The Netherlands (Isolation of Genomic DNA from Mycobacteria Protocol), or according to the source state laboratory’s standard protocol. In brief, *Mtb* cultures were harvested and lysed with lysozyme followed by a SDS/proteinase K mix. Non-nucleic acid cell debris was precipitated using a CTAB/NaCl solution and removed using a chloroform/isoamyl alcohol extraction. Finally, DNA was precipitated using isopropanol. DNA was quantified with the Qubit 2.0 dsDNA Broad Range Assay. Isolate libraries were prepared using the Illumina Nextera XT DNA Library Kit using manual normalization and sequenced on the Illumina MiSeq Platform with v3 Chemistry for 300 bp paired-end reads.

### Data analysis

SNP matrices were produced using a modification of the NASP pipeline [32], with Bowtie2 used for alignment [33], and GATK used for SNP-calling [34], and SNPs being filtered for ten-fold read coverage and 75% read consensus as previously described [28]. Repetitive regions were removed by the NASP pipeline utilizing MUMmer to perform a self-self comparison with a minimum match length of 20. When two compared isolates presented with 30 SNPs or fewer between them during analysis of the pipeline output, those differentiating SNP loci were compared against their alignment’s annotated scaffold genomes in NCBI GenBank (https://www.ncbi.nlm.nih.gov/nuccore/) to identify and discard any SNPs in repetitive regions that were not automatically excluded by the NASP pipeline. Relatedness of isolates was determined by the method developed by Walker et al. [28], with the stepwise 95% prediction interval from the mean rate of change between their paired isolates being used as our baseline. Identification of SNPs among members of clusters, or between isolate pairs, was performed by importing the SNP matrices produced by the NASP pipeline into a database and performing custom SQL queries. Minimum spanning trees were produced for selected clusters with PHYLOViZ 2.0 using goeBURST Full MST [35, 36].

Analysis of the resolving capability of CDC-standard *Mtb* fingerprinting (spoligotyping plus 24 loci MIRU-VNTR typing) was conducted on all 562 fully fingerprinted *Mtb* isolates recorded in the State of Hawaii from 2002 through 2016. Only isolates with the “EAI2_MANILLA” designation in SpolDB4 were utilized as “lineage 1” isolates, as we have previously shown that other spoligotypes with “EAI” designations can span diverse evolutionary lineages [37]. All isolates with “BEIJING” or “BEIJING-LIKE” spoligotypes were placed in “lineage 2.” All isolates with LAM, H, S, T, U, and X spoligotypes were grouped into “lineage 4.” MIRU-VNTR loci were individually analyzed using the Shannon diversity index. Evenness of allelic distribution at each locus was calculated by dividing the Shannon diversity index by the maximum possible Shannon diversity index for that locus, assuming that all alleles could possibly be observed at each locus. Statistical significance of the means of diversity indices for all 24 MIRU-VNTR loci was calculated in Microsoft Excel using the t-Test: Two-Sample Assuming Unequal Variances, with *p*-values < 0.05 being considered significant. Sensitivity and specificity for genetic fingerprinting were calculated using VassarStats Clinical Calculator 1 [38].

Isolates discussed in this paper are identified by their one or two-digit University of Hawaii DNA extraction number. Gene names are presented as annotated in their respective genomes hosted in GenBank (see accession numbers below).

## Results

### Resolution of *Mtb* fingerprinting clusters in Hawaii through whole genome sequencing

Nineteen *Mtb* fingerprinting clusters were either fully (*n* = 15) or partially (*n* = 4) sequenced, with two or more isolates selected for initial sequencing to evaluate the possibility that the fingerprinting cluster represents a direct or recent transmission cluster. Eight of the fingerprinting clusters were determined to represent actual transmission clusters due to isolates in those clusters being separated by fewer than six SNPs. Four clusters were determined to partially represent direct transmission, meaning that while some isolates in the fingerprinting cluster were separated by five or fewer SNPs, other isolates within the fingerprinting cluster were separated by 12 or more SNPs. All sequenced fingerprinting clusters are summarized in Table 1.

### Results of each investigated fingerprinting or epidemiological cluster

The following sections present background information, epidemiological details, and whole genome SNP numbers for each *Mtb* fingerprinting or epidemiological cluster investigated in this study. The clusters are divided into three groups: 1.) Large Clusters Identified by Identical Genetic Fingerprints, 2.) Clusters Identified by Shared Uncommon Spoligotypes, and 3.) Epidemiologically Identified Putative Clusters. Epidemiological details were primarily derived from the chart reviews and reviews of Lanakila TB Clinic Nursing Contact Investigation records that were conducted for a more detailed understanding of transmission. These reviews reinforced several cases where clusters were determined to represent or not represent transmission based on SNP count.

### Large clusters identified by identical genetic fingerprints

These large clusters were identified through CDC-standard genetic fingerprinting (spoligotyping plus 24-loci MIRU-VNTR) of all isolates in Hawaii from 2004 to 2016, plus a selection from 2002, 2003, and 2017. All clusters are detailed in Additional file 1, and their SNP matrices are presented in Table 2.

**Table 2:** SNPs between Sequenced Isolates from Clusters Identified by Identical Genetic Fingerprints

Manila Cluster 1				
	**2**	**9**	**46**	
**2**	X	–	–	
**9**	73	X	–	
**46**	148	135	X	
Beijing Cluster 1				
	**10**	**28**	**29**	**40**
**10**	X	–	–	–
**28**	49	X	–	–
**29**	49	0	X	–
**40**	52	25	25	X

Matrix displaying the number of SNPs between each pair of isolates from Manila Cluster 1 and Beijing Cluster 1

#### Manila cluster 1

**Fig. 1:**
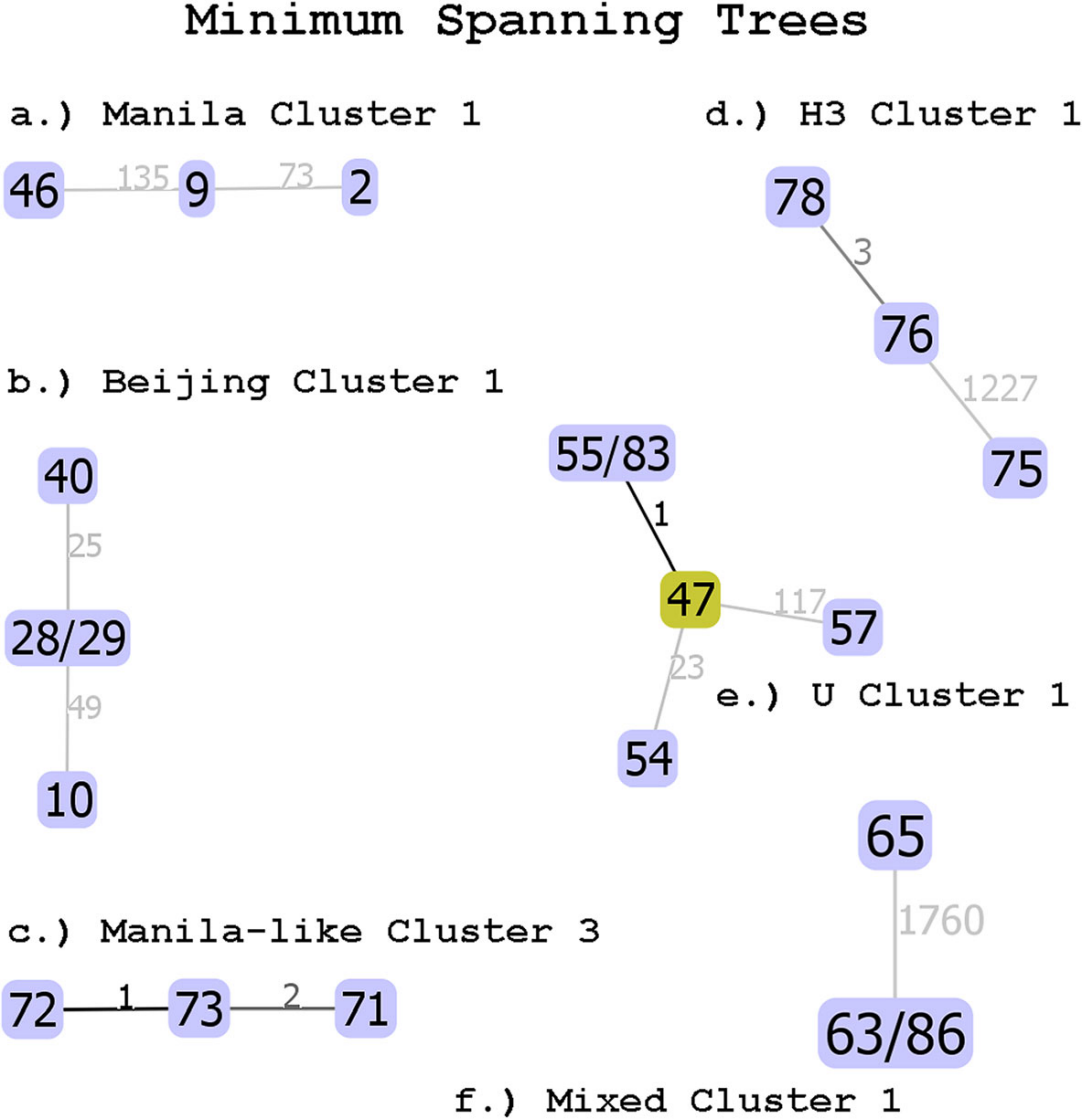
Minimum Spanning Trees for Selected Clusters. Plates a-f present minimum spanning trees for selected putative clusters that contain more than two sequenced isolates. These clusters are discussed individually in following sections. Isolates are identified by their University of Hawaii DNA extraction numbers. The number of SNPs separating each isolate or group of isolates is shown next to each connecting line. Trees shown were determined by PHYLOViZ 2.0 using goeBURST Full MST.

This cluster was composed of 23 isolates spanning 2004–2013. The oldest and newest isolates, plus an intermediate isolate from 2009, were selected for WGS to determine if this fingerprinting cluster might represent an actual transmission cluster. All isolates shared the 677,777,477,413,731 spoligotype and a 254,326,223,432 14A943263217 24-loci MIRU-VNTR fingerprint. The large number of SNPs among isolates in this fingerprinting cluster indicates that it does not represent an actual transmission cluster (Table 2, Fig. 1a). Isolates in this cluster had no epidemiological linkages, reinforcing the WGS-based determination of no comprehensive direct transmission.

#### Manila cluster 2

This cluster was composed of 24 isolates spanning 2004–2013. The oldest and newest isolates (3 and 45, respectively) were selected for WGS to determine if this fingerprinting cluster represents an actual transmission cluster. All isolates shared the 677,777,477,413,771 spoligotype and a 254,326,223,432 14A943263217 24-loci MIRU-VNTR fingerprint. We identified 161 SNPs between the two sequenced isolates, thus allowing us to exclude the possibility that all isolates in this cluster belong to an actual transmission cluster. That result illustrates the need for further WGS fingerprinting to investigate the remaining isolates in the cluster after demonstrating that MIRU-VNTR based clustering is insufficient to conclude that all identically fingerprinting isolates here are actually transmission-linked. Similar to Manila Cluster 1, isolates in this cluster had no epidemiological linkages, reinforcing the WGS-based determination of no comprehensive direct transmission.

#### Beijing cluster 1

This cluster was composed of 11 isolates spanning 2009–2012. The oldest and newest isolates were selected for WGS to determine if this fingerprinting cluster represents an actual transmission cluster. Two intermediate isolates were also sequenced, both to represent possible intermediate transmission isolates and also due to the fact that both patients were originally from the Democratic People’s Republic of Korea (North Korea), and both cases were counted on the same date. All isolates shared the 000000000003771 spoligotype and a 222,325,173,533 445,644,423,328 24-loci MIRU-VNTR fingerprint. The > 20 SNPs displayed by all isolates other than 28 and 29 (which were identical) indicates that this fingerprinting cluster contains both transmission-linked and non-linked isolates (Table 2, Fig. 1b). This fingerprinting cluster revealed no epidemiological linkages, but it is notable that the two isolates that were linked by WGS (separated by no SNPs) were from two retirement-aged, North Korean women.

#### Beijing cluster 2

This cluster was composed of seven Beijing family isolates from 2010 to 2012. All isolates in the cluster were sequenced. All isolates shared the 000000000003771 spoligotype and a 222,325,173,533 445,644,423,326 24-loci MIRU-VNTR fingerprint. All isolates are separated by no SNPs at the 75% read consensus level, indicating that this fingerprinting cluster represents an actual transmission cluster. However, Isolate 35 was distinguished from all other isolates in the cluster by a single SNP in a ~ 100 bp region between a hypothetical protein and a glycosyl hydrolase, which had a read depth of 27x and a 74.4% read consensus (20 reads supporting the SNP and 7 reads supporting the reference), though this SNP was discarded here with a strict read consensus cut-off of 75%. In this fingerprinting cluster (where all isolates were determined to be transmission-linked by WGS) one patient was originally evaluated as a link to another (though he was asymptomatic at the time), and possibly linked to a third case. Of the two possibly linked cases, both were young men from Chuuk (in the Federated States of Micronesia) who entered Hawaii within 5 months of each other.

### Clusters identified by shared uncommon Spoligotypes

All clusters are detailed in Additional file 2, and their SNP matrices are presented in Table 3.

**Table 3:** SNPs between Isolates from Clusters Identified by Shared Uncommon Spoligotypes

Manila-like Cluster 3				
	**71**	**72**	**73**	
**71**	X			
**72**	3	X		
**73**	1	2	X	
H3 Cluster 1				
	**75**	**76**	**78**	**XX**
**75**	X	–	–	–
**76**	1227	X	–	–
**78**	1230	3	X	–
**XX**	NA	NA	NA	X

Matrix displaying the number of SNPs between each pair of isolates from Manila-like Cluster 3 and H3 Cluster 1. With all isolates in Manila-like Cluster 3 separated by only one to three SNPs, this fingerprinting cluster was determined to represent an actual transmission cluster. For H3 Cluster 1, with only three SNPs between isolates 76 and 78, transmission is indicated between them, but not between them and 75. One final isolate has not been sequenced for this project as it was unavailable, and its DNA extraction number has been notated here as “XX”

#### Manila-like cluster 1

This cluster was composed of two isolates from 2011 (21) and 2013 (44) with the Manila-like spoligotype of 600,777,477,413,771 with no SpolDB4 match. Three SNPs were found between the two isolates, indicating that direct or recent transmission is likely. Neither of the cases comprising this fingerprinting cluster were epidemiologically connected to each other.

#### Manila-like cluster 2

This cluster was composed of two isolates from 2011 (30) and 2012 (37) with the Manila-like spoligotype of 677,777,402,003,771 with no SpolDB4 match. Isolate 30 was from the island of Kauai, while 37 was from the island of Hawaii. Four SNPs were found between the two isolates, indicating that transmission is likely, possibly with an intermediate host, or extended incubation period between transmission events. Both patients were from Micronesia, where transmission might have occurred.

#### Beijing cluster 5

This cluster was composed of two isolates from 2008 (74) and 2009 (77), both sharing an uncommon Beijing family spoligotype (000000000003751). Only a single SNP was found between the two isolates, indicating that this fingerprinting cluster likely represents an actual transmission cluster. The two patients in this cluster were niece and uncle.

#### Manila-like cluster 3

This cluster was composed of three isolates from 2002 (71) and 2006 (72 and 73), all sharing a spoligotype with no SpolDB4 match (737777377413771). One to three SNPs were found among the isolates, indicating that this fingerprinting cluster represents an actual transmission cluster (Table 3, Fig. 1c). Two of the isolates belonged to a mother and her son, and both were isoniazid and streptomycin resistant. The third case was caught by an abnormal chest X-ray on entry to the US in 2006, was originally from the Philippines (unlike the mother, who was from Vietnam), and had no drug susceptibility testing results available.

#### H3 cluster 1

This cluster was composed of four isolates with an H3 spoligotype (777777770020771). Isolate 76 was from 2009, and Isolate 78 was from 2010. Isolate 75’s full records were not available in Hawaii, and one additional isolate was not available for sequencing. With only three SNPs between 76 and 78, but over 1000 SNPs among those and the other isolate, this cluster includes both transmission-linked and non-linked isolates (Table 3, Fig. 1d). All four persons in this fingerprinting/epidemiological cluster were from the Republic of the Marshall Islands, though no epidemiological linkage between them could be found. However, multiple members of this cluster reported that they traveled back and forth between Hawaii and the Marshall Islands. Interestingly, a mother in this cluster (76) had a son (75) who also had TB, but the son’s isolate was in the Beijing family instead of H3 and as expected, was separated from the mother’s isolate by over 1000 SNPs.

### Epidemiologically identified putative clusters

These clusters were initially identified as possible transmission clusters by epidemiological investigations instead of by fingerprinting. All clusters are detailed in Additional file 3, and their SNP matrices are presented in Table 4.

**Table 4:** SNPs between Isolates from Epidemiologically Identified Clusters

U Cluster 1					
	**47**	**54**	**57**	**83**	***55***
**47**	X	–	–	–	–
**54**	23	X	–	–	–
**57**	117	120	X	–	–
**83**	1	24	118	X	–
***55***	*1*	*24*	*118*	*0*	*X*
Mixed Cluster 1					
	**63**	**65**	**86**		
**63**	X				
**65**	1761	X			
**86**	0	1762	X		

Matrix displaying the number of SNPs between each pair of isolates from two epidemiologically identified clusters. Isolate 55 (italicized) from Mixed Cluster 2 was added to U Cluster 1 for comparison due to having an identical spoligotype and MIRU-VNTR fingerprint. With only a single SNP separating isolates 55 and 83 from isolate 47, but a larger number of SNPs separating those isolates from isolates 54 and 57, this cluster includes both direct transmission and non-linked isolates. The presence of zero SNPs between isolates 63 and 86 from Mixed Cluster 1 indicates direct transmission. The presence of more than 12 SNPs between isolates 63 and 65 or 65 and 86 indicates that direct transmission between them can be ruled out. This confirms the expected result indicated by their different spoligotyping lineages

#### Manila cluster 3

This cluster was composed of two Manila family isolates from patients from the Philippines (53 from 2015 and 61 from 2016). We identified 90 SNPs between the two isolates, allowing direct transmission to be ruled out. Initially (prior to WGS analysis), this fingerprinting cluster appeared to represent a transmission cluster due to being composed of an uncle and nephew, though WGS later disproved that possibility.

#### Manila cluster 4

This cluster was composed of two Manila family isolates from a husband and wife, both originally from the Philippines (51 and 59). Zero SNPs separated the two isolates, indicating direct transmission.

#### Manila cluster 5

This cluster was composed of two Manila family isolates from two siblings living in the same city, both originally from the Philippines, who were diagnosed 2 years apart (50 and 62). One isolate was multiple drug resistant (MDR), while the other isolate was pan-susceptible to antibiotics, initially suggesting to TB controllers that these two isolates were not transmission-linked. The 192 SNPs found between the two isolates further excluded the possibility of direct transmission.

#### Manila cluster 6

This cluster was composed of two Manila family isolates from an aunt and nephew, both originally from the Philippines (84 and 60). One isolate was isoniazid and pyrazinamide resistant while the other was pan-susceptible. The 229 SNPs identified between the two isolates allowed direct transmission to be further ruled out.

#### Beijing cluster 3

This cluster was composed of two Beijing family isolates from a husband and wife, one of whom was US-born and the other (the index patient) was from the Republic of the Marshall Islands (RMI) (58 and 85). The three SNPs identified between the two isolates supported the epidemiological assessment that direct or recent transmission had occurred.

#### Mixed cluster 2

This cluster was included in this study prior to CDC fingerprinting being conducted. Isolate 56 was from a retirement-aged man from the RMI who had entered the US 1 month prior to his case being documented. Isolate 55 came from a young woman, also from the RMI, who entered the US roughly 15 years prior, and had a suspicious chest X-ray (but negative follow-up X-ray) 2 years prior to her diagnosis, and then was determined to have TB when examined as a contact to the patient providing isolate 56. However, the two isolates had different spoligotypes (Beijing for 56 and U for 55), and the 1153 SNPs between them further confirmed that transmission of these strains had not occurred between these two patients.

#### U cluster 1

U Cluster 1 is perhaps the most interesting epidemiologically identified cluster for multiple reasons (Fig. 1e). It was identified not by contact-investigation in Hawaii, but by the US Centers for Disease Control and Prevention (CDC), who notified the State of Hawaii TB Control Program of it. The TB Control Program reviewed the cases, but was unable to find any epidemiological linkages among them**.** All isolates shared the same uncommon, unclassified “U” SpolDB4 spoligotype designation (777777760000000) and the same 24-loci MIRU-VNTR fingerprint (223,325,143,322 242,324,223,422). All cases were young men from either the Federated States of Micronesia or the Marshall Islands (Additional file 4). We further noted that isolate 55 from Mixed Cluster 2 shared the same spoligotype and MIRU-VNTR fingerprint as the isolates in this cluster, and thus we included it in our comparison. WGS identified likely direct transmission between two patients in this fingerprinting/outbreak notification cluster (isolates 47 and 83), with only one SNPs between them, and 0–1 SNPs between them and the addition from Mixed Cluster 2, isolate 55 (Fig. 1). However, the SNP numbers between those three isolates and the remaining two isolates in the fingerprinting cluster (54 and 57) are considerably higher, indicating that some isolates in this cluster are linked by direct transmission, while others are not. Thus, while this outbreak notification cluster does contain transmission-linked isolates, the linkage does not extend to all isolates in the notification. Furthermore, it appears that one additional isolate (55) is linked to this cluster, despite not being included in the notification.

#### Mixed cluster 1

This putative cluster is composed of three isolates from the island of Maui, two of which are in the Beijing family (63 and 86) and one of which is in the Manila family (65). The two Manila family isolates represent direct transmission with zero SNPs separating them, while the Beijing family isolate is not related, with 1700+ SNPs separating it from the Manila family isolates (Table 4, Fig. 1f). Although detailed epidemiological information was not available for this cluster, the two Manila family isolates being separated by only a single SNP indicates that this epidemiologically identified cluster represents a partial transmission cluster.

#### Manila cluster 7

This cluster was composed of two Manila family isolates from a grandfather and grandson, both originally from the Philippines and who both lived together in Hawaii (48 and 49). Both cases were counted by the State of Hawaii on the same month, but the grandfather’s isolate was MDR, while the grandson’s isolate was pan-susceptible. The 142 SNPs between the two isolates confirmed that the two cases were not the result of direct transmission.

#### Beijing cluster 4

This cluster was composed of two Beijing family isolates from a juvenile male and a middle-aged man from the Marshall Islands (whose relationship with the juvenile is unclear, but may have served as his guardian at some point) (52 and 64). The older man previously had TB and was treated in the Marshall Islands, but his tuberculin skin test (TST) showed a negative reaction upon entering Hawaii. Later, the juvenile, living in a shelter at the time, was treated for TB and the man was identified as a contact, at which time the man produced a suspicious chest X-ray and a positive T-SPOT.TB interferon gamma release assay (IGRA) test. However, his TST and QuantiFERON-TB IGRA test were negative, in addition to no acid-fast bacilli being found in his sputum and his sputum culture growing no bacteria, so he was not treated at that time. Regardless, he produced a culture-positive sputum sample 16 months later, at which time the juvenile was also re-identified as a contact. Despite this complexity, zero SNPs were found between the two isolates, unambiguously indicating direct transmission.

### Sensitivity and specificity of standard *Mtb* fingerprinting

Comparing the ability of WGS and standard *Mtb* fingerprinting to resolve the ten epidemiologically identified *Mtb* clusters in this study showed that fingerprinting indicated that eight of the ten epidemiological clusters were genetic clusters, while WGS indicated that only three of the ten epidemiological clusters were genetic clusters (Additional file 5). Standard fingerprinting was unable to resolve any of the clusters indicated by WGS. If WGS is designated as the “gold standard” for *Mtb* typing, then the sensitivity *Mtb* fingerprinting for identifying actual transmission clusters was found to be 100% (95% CI, 31.0–100%), while its specificity was only 28.6% (95% CI, 5.1–69.7%).

### MIRU-VNTR resolution of Beijing and Manila family clusters

**Fig. 2:**
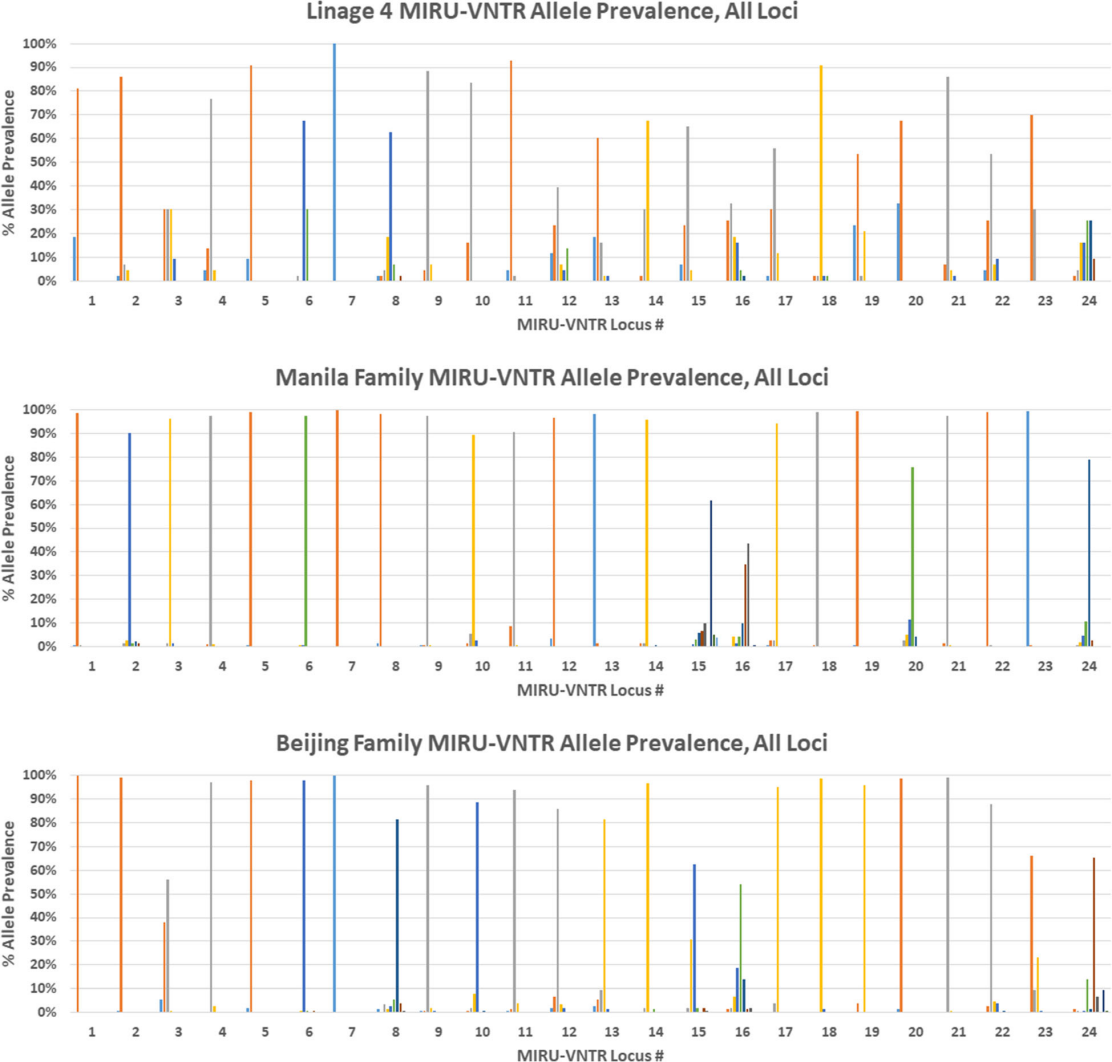
Comparison of MIRU-VNTR Allele Prevalence by Lineage and Family. Each numbered group of bars represents one locus used in 24 loci MIRU-VNTR typing of *Mtb*. Vertical bars represent the percentage of all alleles at each locus that each allele comprises. The reduced allelic evenness demonstrated by the Beijing and Manila families relative to lineage 4 at most loci is readily observed

**Fig. 3:**
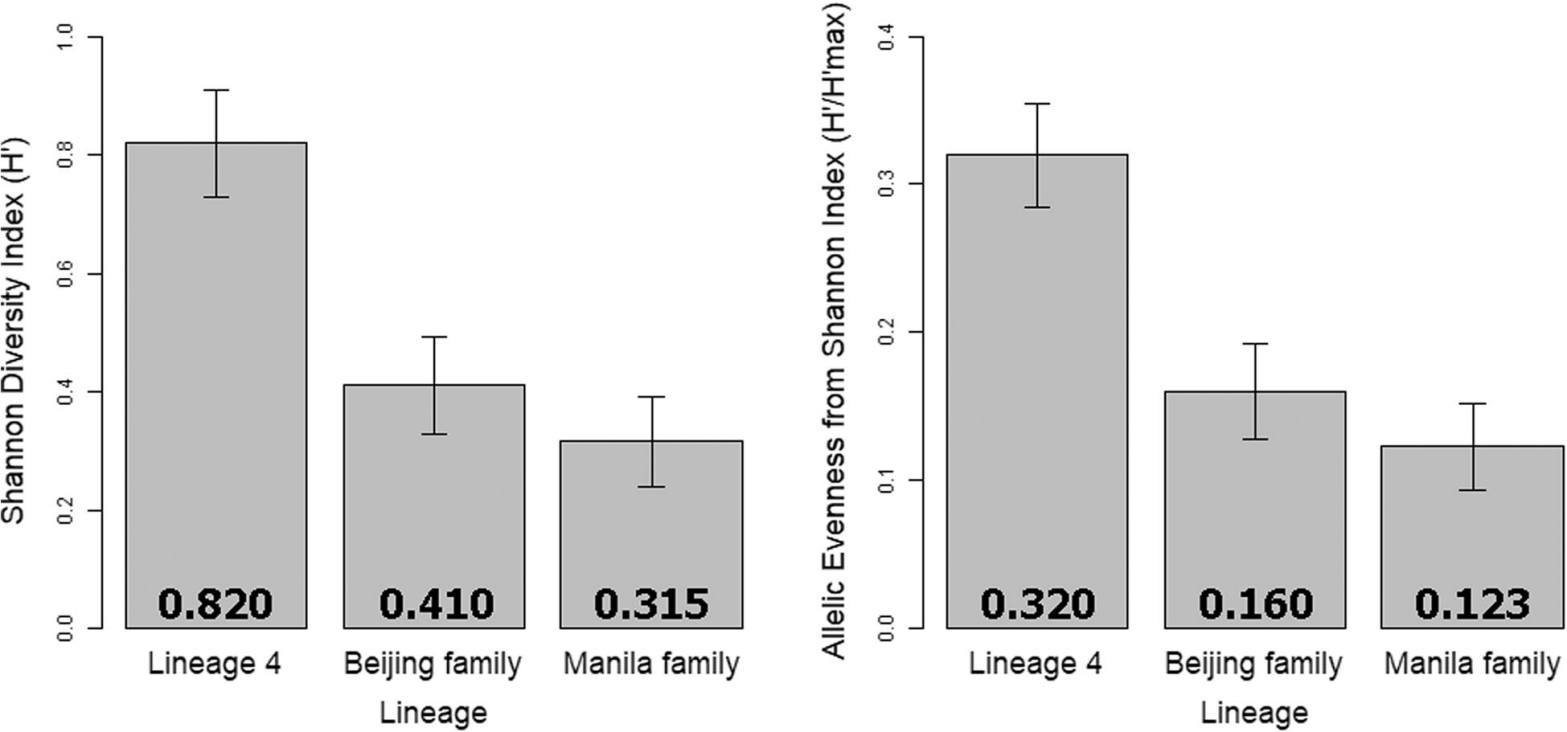
Mean Shannon Diversity Index and Evenness Values for 24 MIRU-VNTR Loci, by Lineage. The Shannon diversity index and evenness values of the Beijing and Manila families are statistcally significantly lower than lineage 4 for both metrics at the *p* = 0.005 level, but the Beijing and Manila families are not significantly different from each other at the *p* = 0.05 level for either metric. Error bars represent 95% confidence intervals

Of the 562 isolates in this study that were fully fingerprinted with 24-loci MIRU-VNTR and that had no loci that failed to sequence, 369 were in the Manila family (lineage 1), 150 were in the Beijing family (lineage 2), and 43 were in the various spoligotyping clades of lineage 4. (All of Hawaii’s *Mtb* isolates were typed with 24 loci MIRU-VNTR fingerprinting from 2009 onwards, but most isolates prior to 2009 were only typed with 12-loci MIRU-VNTR typing and thus were not included in this analysis.) Analysis of MIRU-VNTR allele distribution over the full set of 24 loci indicated reduced Shannon diversity indices and Shannon evenness values of MIRU-VNTR allele distribution in the Beijing and Manila families (lineages 2 and 1) compared to lineage 4 (Additional file 6). This reduced evenness is readily apparent from the histogram of allele distributions presented in Fig. 2. The Beijing family displayed significantly lower average Shannon diversity indices (*p* ≤ 0.002) and Shannon evenness values (*p* ≤ 0.002) over the full set of 24 loci than lineage 4 (Fig. 3). Likewise, the Manila family displayed significantly lower average Shannon diversity indices (*p* ≤ 0.001) and Shannon evenness values (*p* ≤ 0.001) over the full set of 24 loci than lineage 4. However, the Beijing and Manila families were not significantly different from each other by that index (*p* = 0.4).

### Genes containing intra-cluster SNPs

Every putative cluster that was determined to represent an actual transmission cluster and whose isolates were separated by at least one SNP was examined to determine which genes hosted the intra-cluster mutations. Additional file 7 displays these genes, their mutations, and their TubercuList annotations and descriptions. SNPs from repetitive PPE and PE-PGRS family genes were discarded as questionable SNPs (possibly resulting from alignment errors) in this study because we did not confirm individual SNPs with PCR assays or Sanger sequencing.

## Discussion

This work demonstrated that established standard molecular fingerprinting methods for *Mtb* (spoligotyping plus 24-loci MIRU-VNTR typing) are insufficient for epidemiological investigation of TB in Hawaii. Our study is not alone in such findings. One study that utilized 1999 consecutive *MTb* isolates processed by a laboratory in the English Midlands from 2012 to 2015 identified that the performance of MIRU-VNTR profiles for identifying genomic relatedness in *Mtb* differed by lineage [15]. Notably, when they modeled the number of SNPs between paired isolates assuming a linear relationship over 1–3 MIRU-VNTR locus differences, they found that while paired lineage 4 isolates with identical MIRU-VNTR profiles displayed a median of 10 SNPs, lineages 1 and 2 displayed 122 and 159 SNPs, respectively. However, this study also showed that the number of pairwise SNPs between isolates was significantly higher when one or both isolates were from a recent immigrant, suggesting that the study’s specific conclusions partially represented trends in domestic versus foreign transmission associated with different lineages. Regardless, it further illustrates the necessity of WGS over MIRU-VNTR for investigation of Mtb transmission.

With WGS serving as our “gold standard,” we demonstrated the specificity of CDC-standard fingerprinting (spoligotyping plus MIRU-VNTR) in our geographic region with high levels of Beijing and Manila family *Mtb* to be only 28.6% (Additional file 5). Such a low level provides clinicians and epidemiologists with very low confidence that a purported transmission cluster identified by standard fingerprinting represents an actual transmission cluster. Note that these data are not intended to propose that WGS be considered the gold standard for *Mtb* epidemiological analysis; rather, they are intended to illustrate how high prevalence of certain *Mtb* families exposes shortcomings in presently-employed *Mtb* genetic fingerprinting methods. However, although IS*6110* has previously been considered the “gold standard” for *Mtb* molecular epidemiology, isolates with as many as 130 SNPs between them have been shown to have identical IS*6110* fingerprints, adding support that WGS has become the de-facto “gold standard” for *Mtb* molecular epidemiology [39, 40].

Our previous work illustrated that even with the full set of 24 MIRU-VNTR loci, potential Beijing and Manila family transmission clusters are poorly resolved by this method of fingerprinting [29]. Here, we identified that MIRU-VNTR’s lack of resolving ability results from the Beijing and Manila families both being characterized by a greater number of loci that are dominated by either one allele or a small set of alleles than lineage 4. While the Shannon diversity index itself does not indicate how much of its diversity is derived from allelic richness versus allelic evenness, evenness can be easily calculated using values from the Shannon diversity index. Figure 3 shows that most of the reduction in Shannon diversity demonstrated by the Beijing and Manila families is due to a decrease in allelic evenness instead of a decrease in allelic diversity. However, it should be noted that lineage 4 contains multiple major clades, compared to one clade each for lineages 1 and 2, and thus higher allelic evenness should generally be expected from lineage 4 overall. Regardless, this work illustrates why MIRU-VNTR fingerprinting is less effective at identifying actual transmission when applied to Beijing and Manila family isolates.

These data help demonstrate why CDC-standard molecular fingerprinting of *Mtb* is insufficient for areas of the world where the Beijing and Manila families are dominant. Thus, this study investigated in detail the ability of whole genome sequencing-based analysis to compensate for MIRU-VNTR’s shortcoming by resolving fingerprinting-derived clusters from those two families in order to identify actual transmission.

### Combining epidemiology with whole genome sequencing for cluster resolution

Of the 19 possible transmission clusters we investigated, definitive verdicts of recent transmission, partial transmission, or non-transmission were reached for all clusters. Epidemiological investigation was used to further strengthen or disprove the determinations of transmission or non-transmission. Although WGS analysis was able to disprove the apparent transmission that was initially suspected based on epidemiological connections for several apparent clusters, there were no cases where epidemiological information was sufficient to call WGS-derived transmission determinations into question.

### Genes containing cluster-informative SNPs

In order to explore which genes could be experiencing rapid mutation and producing the SNPs that distinguished isolates within individual transmission clusters, isolates from those clusters were aligned against GenBank genome CP003248.2, which was selected due to its manually-curated annotation at TubercuList. These informative SNPs that distinguished isolates in actual transmission clusters are contained in a broad range of genes (Additional file 7). The genes identified in this study differ from those identified by a previous study examining an outbreak in San Francisco with the H1 spoligotype [27]. The genes where intra-cluster SNPs were located did not appear to demonstrate any lineage association, and included an ATPase, an ABC transporter membrane protein, a PHOH-like protein PhoH2 phosphate starvation-inducible protein, a PSIH-like sequence-specific RNA helicase, an RNAse, and several hypothetical proteins, among others [41, 42].

### Determining isolate relatedness through whole genome sequencing

The selection of cut-off points for a SNP’s required read coverage and read consensus (allele frequency) are of interest for developing a system for applied WGS epidemiology. Previous studies have required 75% read consensus or 10x read coverage and 80% read consensus, and found a mutation rate of ~ 0.5 SNPs per genome per year and 0.4 SNPs per genome per year [16, 28]. At the extreme ends of range proposed by Walker et al. for identifying transmission-linked or possibly linked isolates (0–1 SNPs and 6–12 SNPS), this information may suggest to tuberculosis controllers whether two isolates were likely the result of recent, direct transmission, or whether the transmission occurred in the more distant past (allowing time for divergent accumulation of SNPs in each infection) or through an intermediate host [28]. However, with several transmission clusters investigated in this work displaying 3–4 SNPs distinguishing their isolates, we cannot propose whether they represent direct transmission or not – only recent transmission.

## Conclusion

This project demonstrated use of whole genome sequencing to successfully overcome the Beijing and Manila families’ current fingerprinting difficulties, which have been a persistent problem for State of Hawaii tuberculosis control efforts. We identified why even 24-loci MIRU-VNTR fingerprinting fails to effectively resolve Beijing and Manila family clusters, and illustrated the advantage and necessity of utilizing WGS for molecular epidemiology in this region. As we continue to characterize the epidemiology of tuberculosis in Hawaii, more isolates from the largest Beijing and Manila family fingerprinting clusters will be sequenced to provide a more complete picture of their transmission.

## Additional files

Additional file 1: Isolates Selected for WGS from Clusters Identified by Identical Genetic Fingerprints. This table summarizes the isolates that were selected for WGS from clusters that were initially identified by their shared genetic fingerprints. (DOCX 14 kb)

Additional file 2: Clusters Identified by Shared Uncommon Spoligotypes. This table summarizes the isolates that were selected for WGS from clusters that were initially identified by their shared uncommon spoligotypes. (DOCX 13 kb)

Additional file 3: Epidemiologically Identified Clusters. This table summarizes the isolates that were selected for WGS from clusters that were initially identified by epidemiology. (DOCX 14 kb)

Additional file 4: U Cluster 1 Demographics. This table displays the demographics of the patients providing isolates from U Cluster 1. (DOCX 14 kb)

Additional file 5: Comparison of the Sensitivity and Specificity of Standard *Mtb* Fingerprinting against Whole Genome Sequencing (WGS). This “2 × 2 Table” illustrates how the sensitivity and specificity of standard *Mtb* fingerprinting for the epidemiologically-identified clusters in this study were calculated. (DOCX 12 kb)

Additional file 6: Comparison of Individual MIRU-VNTR Loci through the Shannon Diversity Index and Shannon Evenness. This table contains the Diversity Shannon Diversity Index and Shannon Evenness values for each of the 24 standard MIRU-VNTR loci. (DOCX 13 kb)

Additional file 7: Genes Containing Intra-cluster SNPs. This table displays the genes and mutation sites where SNPs that distinguished isolates within clusters were found. (DOCX 14 kb)

## Abbreviations

CDC: Centers for Disease Control and Prevention
MIRU-VNTR: Mycobacterial Interspersed Repetitive Units-Variable Number Tandem Repeats
*Mtb*: *Mycobacterium tuberculosis*
PE-PGRS: Proline-glutamic Acid - Polymorphic GC-Rich Sequence
PPE: Proline-Proline-glutamic Acid
PPV: Positive Predictive Value
SNP: Single Nucleotide Polymorphism
TB: Tuberculosis
WGS: Whole Genome Sequencing
WHO: World Health Organization
