# Supplementary material for: Genomic sequencing is required for identification of tuberculosis transmission in Hawaii
Source: BMC Infect Dis. 2018 Dec 3;18:608. doi: 10.1186/s12879-018-3502-1 (PMC6276198; doi:10.1186/s12879-018-3502-1)
Supplement: Supplementary file 1 — Isolates Selected for WGS from Clusters Identified by Identical Genetic Fingerprints. This table summarizes the isolates that were selected for WGS from clusters that were initially identified by their shared genetic fingerprints. (DOCX 14 kb) [file 12879_2018_3502_MOESM1_ESM.docx]

**Additional File 1:**

**Isolates Selected for WGS from Clusters Identified by Identical Genetic Fingerprints**

| DNA # | City | Country | Birth Year | Arrival Date | Count Date | Cluster |
| --- | --- | --- | --- | --- | --- | --- |
| 2 | 3 | Philippines | - | - | 3/1/2004 | Manila Cluster 1 |
| 9 | 8 | Philippines | - | - | 4/1/2009 | Manila Cluster 1 |
| 46 | 9 | Philippines | - | - | 6/1/2013 | Manila Cluster 1 |
| 3 | 1 | Philippines | - | - | 5/1/2004 | Manila Cluster 2 |
| 45 | 10 | Philippines | - | - | 6/1/2013 | Manila Cluster 2 |
| 10 | 1 | Chuuk | - | July 2008 | 5/1/2009 | Beijing Cluster 1 |
| 28 | 7 | N. Korea | - | 1969 | 12/1/2010 | Beijing Cluster 1 |
| 29 | 11 | N. Korea | - | 1975 | 12/1/2010 | Beijing Cluster 1 |
| 40 | 2 | RMI | - | April 2011 | 4/1/2012 | Beijing Cluster 1 |
| 16 | 1 | Chuuk, FSM | 1989 | July 2008 | 8/1/2010 | Beijing Cluster 2 |
| 22 | 1 | Chuuk, FSM | 1994 | May 2010 | 3/1/2011 | Beijing Cluster 2 |
| 35 | 4 | Chuuk, FSM | 1992 | 2004 | 2/1/2012 | Beijing Cluster 2 |
| 79 | 1 | FSM | 1988 | Jan. 1995* | 9/1/2010 | Beijing Cluster 2 |
| 80 | 2 | Chuuk, FSM | 1976 | 2004 | 1/1/2010 | Beijing Cluster 2 |
| 81 | 1 | Chuuk, FSM | 1989 | Jan. 2007 | 6/1/2011** | Beijing Cluster 2 |
| 82 | 1 | Chuuk, FSM | 1987 | June 2007 | 2/1/2012 | Beijing Cluster 2 |

List of the University of Hawaii DNA extraction numbers (DNA #), encoded city numbers for HIPAA de-identification, countries of origin, years of birth, dates of arrival in the USA, Hawaii DOH case-counted dates, and cluster names for all isolates initially selected due to their shared genetic fingerprints. *Arrived in Hawaii in 2000. **Initially evaluated as a contact to 79/10L8839. Abbreviations are as follows – RMI: Republic of the Marshall Islands, FSM: Federated States of Micronesia.
