# Supplementary material for: Genomic sequencing is required for identification of tuberculosis transmission in Hawaii
Source: BMC Infect Dis. 2018 Dec 3;18:608. doi: 10.1186/s12879-018-3502-1 (PMC6276198; doi:10.1186/s12879-018-3502-1)
Supplement: Supplementary file 2 — Clusters Identified by Shared Uncommon Spoligotypes. This table summarizes the isolates that were selected for WGS from clusters that were initially identified by their shared uncommon spoligotypes. (DOCX 13 kb) [file 12879_2018_3502_MOESM2_ESM.docx]

**Additional File 2:**

**Clusters Identified by Shared Uncommon Spoligotypes**

| DNA # | City | Country | Count Date | Cluster Name |
| --- | --- | --- | --- | --- |
| 21 | 5 | FSM | 2/1/2011 | **Manila-like Cluster 1** |
| 44 | 1 | FSM | 2/1/2013 | **Manila-like Cluster 1** |
| 30 | 13 | USA | 4/1/2011 | **Manila-like Cluster 2** |
| 37 | 14 | RMI | 4/1/2012 | **Manila-like Cluster 2** |
| 74 | 1 | Micronesia | 11/1/2008 | **Beijing Cluster 5** |
| 77 | 1 | Micronesia | 4/1/2009 | **Beijing Cluster 5** |
| 71 | 1 | Vietnam | 5/1/2002 | **Manila-like Cluster 3** |
| 72 | 1 | USA | 2/1/2006 | **Manila-like Cluster 3** |
| 73 | 15 | Philippines | 4/1/2006 | **Manila-like Cluster 3** |
| 75 | - | RMI | - | **H3 Cluster 1** |
| 76 | 1 | RMI | 4/1/2009 | **H3 Cluster 1** |
| 78 | 1 | RMI | 1/1/2010 | **H3 Cluster 1** |
| N/A | 1 | RMI | 3/1/2007 | **H3 Cluster 1** |

Listing of the DNA Extraction Numbers (DNA #), encoded city numbers, countries, and Hawaii DOH case count dates of all isolates from possible clusters identified by their uncommon spoligotypes. 07L2406 has not yet been sequenced for this project, and its DNA extraction number has been identified with “XX.” Abbreviations are as follows – RMI: Republic of the Marshall Islands, FSM: Federated States of Micronesia.
