# Supplementary material for: Genomic sequencing is required for identification of tuberculosis transmission in Hawaii
Source: BMC Infect Dis. 2018 Dec 3;18:608. doi: 10.1186/s12879-018-3502-1 (PMC6276198; doi:10.1186/s12879-018-3502-1)
Supplement: Supplementary file 3 — Epidemiologically Identified Clusters. This table summarizes the isolates that were selected for WGS from clusters that were initially identified by epidemiology. (DOCX 14 kb) [file 12879_2018_3502_MOESM3_ESM.docx]

**Additional File 3:**

**Epidemiologically Identified Clusters**

| DNA # | City | Country | Count Date | Spoligotype | Cluster Name |
| --- | --- | --- | --- | --- | --- |
| 53 | 5 | Philippines | 7/1/2015 | Manila | Manila Cluster 3 |
| 61 | 8 | Philippines | 10/1/2016 | Manila | Manila Cluster 3 |
| 51 | 5 | Philippines | 9/1/2014 | Manila | Manila Cluster 4 |
| 59 | 5 | Philippines | 12/1/2015 | Manila | Manila Cluster 4 |
| 50 | 5 | Philippines | 6/1/2014 | Manila | Manila Cluster 5 |
| 62 | 5 | Philippines | 1/1/2016 | Manila | Manila Cluster 5 |
| 84 | 2 | Philippines | 5/1/2013 | Manila | Manila Cluster 6 |
| 60 | 2 | Philippines | 1/1/2016 | Manila | Manila Cluster 6 |
| 58 | 1 | USA/HI | 12/1/2015 | Beijing | Beijing Cluster 3 |
| 85 | 1 | RMI | 6/1/2013 | Beijing | Beijing Cluster 3 |
| 56 | 1 | RMI | 9/1/2015 | Beijing | Mixed Cluster 2 |
| 55 | 1 | RMI | 9/1/2015 | U | Mixed Cluster 2 |
| 47 | 1 | Chuuk | 10/1/2013 | U | U Cluster 1 |
| 54 | 1 | Chuuk | 7/1/2015 | U | U Cluster 1 |
| 57 | 1 | Chuuk | 8/1/2015 | U | U Cluster 1 |
| 83 | 1 | - | 4/1/2013 | U | U Cluster 1 |
| 63 | 12 | - | 2/1/2016 | Beijing | Mixed Cluster 1 |
| 65 | 8 | - | 7/1/2016 | Manila | Mixed Cluster 1 |
| 86 | 12 | - | 9/1/2016 | Beijing | Mixed Cluster 1 |
| 48 | 1 | Philippines | 2/1/2014 | Manila | Manila Cluster 7 |
| 49 | 1 | Philippines | 2/1/2014 | Manila | Manila Cluster 7 |
| 64 | 2 | RMI | 6/1/2016 | Beijing | Beijing Cluster 4 |
| 52 | 4 | RMI | 2/1/2015 | Beijing | Beijing Cluster 4 |

Listing of the DNA Extraction Numbers (DNA #), encoded city numbers, countries, Hawaii DOH case count dates, and spoligotypes of all isolates from epidemiologically identified clusters. Abbreviations are as follows – RMI: Republic of the Marshall Islands, HI: Hawaii.
