# Supplementary material for: Genomic sequencing is required for identification of tuberculosis transmission in Hawaii
Source: BMC Infect Dis. 2018 Dec 3;18:608. doi: 10.1186/s12879-018-3502-1 (PMC6276198; doi:10.1186/s12879-018-3502-1)
Supplement: Supplementary file 4 — U Cluster 1 Demographics. This table displays the demographics of the patients providing isolates from U Cluster 1. (DOCX 14 kb) [file 12879_2018_3502_MOESM4_ESM.docx]

**Additional File 4:**

**U Cluster 1 Demographics**

| **DNA #** | **City** | **Country** | **Age** | **Arrival Date** | **Count Date** |
| --- | --- | --- | --- | --- | --- |
| **47** | 1 | Chuuk | 15-20 | 2012 | 10/1/13 |
| **54** | 1 | Chuuk | 15-20 | 2008 | 7/1/15 |
| **57** | 1 | Chuuk | 20-25 | 2010 | 8/1/15 |
| **83** | 1 | - | - | - | 4/1/13 |
| **55** | 1 | RMI | 20-25 | 2000 | 9/1/15 |

List of the DNA extraction numbers (DNA #), encoded city numbers, countries of origin, ages, arrival dates in the USA, and Hawaii DOH case count dates for all isolates from U Cluster 1. This cluster was identified by the CDC as an outbreak cluster, despite no epidemiological connections from Hawaii being identified. Isolate 55/15RF6749 from Mixed Cluster 2 was added to this cluster for comparison due to having an identical spoligotype and MIRU-VNTR fingerprint.
