# Supplementary material for: Genomic sequencing is required for identification of tuberculosis transmission in Hawaii
Source: BMC Infect Dis. 2018 Dec 3;18:608. doi: 10.1186/s12879-018-3502-1 (PMC6276198; doi:10.1186/s12879-018-3502-1)
Supplement: Supplementary file 5 — Comparison of the Sensitivity and Specificity of Standard Mtb Fingerprinting against Whole Genome Sequencing (WGS). This “2 × 2 Table” illustrates how the sensitivity and specificity of standard Mtb fingerprinting for the epidemiologically-identified clusters in this study were calculated. (DOCX 12 kb) [file 12879_2018_3502_MOESM5_ESM.docx]

**Additional File 5:**

**Comparison of the Sensitivity and Specificity of Standard *Mtb* Fingerprinting against Whole Genome Sequencing (WGS)**

|  | **Clustered by WGS** | **Resolved by WGS** |
| --- | --- | --- |
| **Clustered by Fingerprinting** | 3 | 5 |
| **Resolved by Fingerprinting** | 0 | 2 |

2x2 table of the 10 epidemiologically-identified clusters in this study, where each number represents the number of clusters meeting the listed criteria. With WGS designated as the “gold standard,” this table allows us to calculate the sensitivity of standard *Mtb* fingerprinting for identifying actual transmission clusters to be 100% (CI95 31.0% - 100%), while its specificity is only 28.6% (CI95 5.1% - 69.7%). Sensitivity and specificity were calculated using VassarStats Clinical Calculator 1 (<http://vassarstats.net/clin1.html>). Three clusters were determined to be transmission clusters by both standard fingerprinting and WGS (Manila Cluster 4, Beijing Cluster 3, and Beijing Cluster 4). Two epidemiological clusters were resolved by both standard fingerprinting and WGS (Mixed Clusters 1 and 2). Five epidemiological clusters that were determined to be transmission clusters by standard fingerprinting were resolved by WGS (Manila Cluster 3, Manila Cluster 5, Manila Cluster 6, U Cluster 1, and Manila Cluster 7). No epidemiological clusters that were determined to be actual transmission clusters by WGS were determined not to be transmission clusters by standard fingerprinting.
