# Supplementary material for: Genomic sequencing is required for identification of tuberculosis transmission in Hawaii
Source: BMC Infect Dis. 2018 Dec 3;18:608. doi: 10.1186/s12879-018-3502-1 (PMC6276198; doi:10.1186/s12879-018-3502-1)
Supplement: Supplementary file 6 — Comparison of Individual MIRU-VNTR Loci through the Shannon Diversity Index and Shannon Evenness. This table contains the Diversity Shannon Diversity Index and Shannon Evenness values for each of the 24 standard MIRU-VNTR loci. (DOCX 13 kb) [file 12879_2018_3502_MOESM6_ESM.docx]

**Additional File 6:**

**Comparison of Individual MIRU-VNTR Loci through the Diversity Shannon Diversity Index and Shannon Evenness**

|  | **Shannon Diversity Index (H')** | | | **Evenness (H'/H_max_)** | | |
| --- | --- | --- | --- | --- | --- | --- |
| **Locus** | **Lineage 4** | **Beijing** | **Manila** | **Lineage 4** | **Beijing** | **Manila** |
| **1** | 0.480 | 0.000 | 0.081 | 0.187 | 0.000 | 0.032 |
| **2** | 0.545 | 0.040 | 0.489 | 0.213 | 0.016 | 0.191 |
| **3** | 1.306 | 0.882 | 0.203 | 0.509 | 0.344 | 0.079 |
| **4** | 0.763 | 0.123 | 0.138 | 0.298 | 0.048 | 0.054 |
| **5** | 0.309 | 0.098 | 0.066 | 0.121 | 0.038 | 0.026 |
| **6** | 0.715 | 0.120 | 0.150 | 0.279 | 0.047 | 0.059 |
| **7** | 0.000 | 0.000 | 0.019 | 0.000 | 0.000 | 0.007 |
| **8** | 1.196 | 0.812 | 0.102 | 0.466 | 0.316 | 0.040 |
| **9** | 0.438 | 0.218 | 0.147 | 0.171 | 0.085 | 0.057 |
| **10** | 0.444 | 0.454 | 0.471 | 0.173 | 0.177 | 0.184 |
| **11** | 0.297 | 0.278 | 0.347 | 0.116 | 0.108 | 0.135 |
| **12** | 1.560 | 0.580 | 0.162 | 0.608 | 0.226 | 0.063 |
| **13** | 1.088 | 0.700 | 0.102 | 0.424 | 0.273 | 0.040 |
| **14** | 0.715 | 0.169 | 0.228 | 0.279 | 0.066 | 0.089 |
| **15** | 0.947 | 0.923 | 1.206 | 0.369 | 0.360 | 0.470 |
| **16** | 1.553 | 1.373 | 1.353 | 0.605 | 0.535 | 0.528 |
| **17** | 1.025 | 0.174 | 0.276 | 0.400 | 0.068 | 0.107 |
| **18** | 0.438 | 0.071 | 0.052 | 0.171 | 0.028 | 0.020 |
| **19** | 1.089 | 0.168 | 0.034 | 0.424 | 0.065 | 0.013 |
| **20** | 0.631 | 0.071 | 0.913 | 0.246 | 0.028 | 0.356 |
| **21** | 0.545 | 0.040 | 0.135 | 0.213 | 0.016 | 0.053 |
| **22** | 1.233 | 0.514 | 0.066 | 0.481 | 0.201 | 0.026 |
| **23** | 0.613 | 0.869 | 0.034 | 0.239 | 0.339 | 0.013 |
| **24** | 1.740 | 1.171 | 0.798 | 0.678 | 0.456 | 0.311 |

Shannon Diversity Index and Evenness computed for all 24 MIRU-VNTR loci for all MTB isolates grouped into Lineage 4, Beijing family, and Manila family sets.
