# Supplementary material for: Genomic sequencing is required for identification of tuberculosis transmission in Hawaii
Source: BMC Infect Dis. 2018 Dec 3;18:608. doi: 10.1186/s12879-018-3502-1 (PMC6276198; doi:10.1186/s12879-018-3502-1)
Supplement: Supplementary file 7 — Genes Containing Intra-cluster SNPs. This table displays the genes and mutation sites where SNPs that distinguished isolates within clusters were found. (DOCX 14 kb) [file 12879_2018_3502_MOESM7_ESM.docx]

**Additional File 7:**

**Genes Containing Intra-cluster SNPs**

| **Cluster and Locus ID** | **Reference Allele** | **Isolate Alleles** | | **Tuberculist Gene** | **Gene Name** |
| --- | --- | --- | --- | --- | --- |
| **Manila-like Cluster 1** | H37Rv | 21 | 44 |  |  |
| CP003248.2::1000850 | T | T | C | Rv0897c | oxidoreductase |
| CP003248.2::1224186 | G | G | A | Rv1095 | PHOH-like protein PhoH2 |
| CP003248.2::3980547 | C | C | T | Rv3540c | lipid-transfer protein or keto acyl-CoA thiolase Ltp2 |
| **Manila-like Cluster 2** | H37Rv | 30 | 37 |  |  |
| CP003248.2::1052423 | T | T | C | Rv0942 | hypothetical protein |
| CP003248.2::1097833 | A | A | T | Rv0982 | two-component system sensor histidine kinase MprB |
| CP003248.2::2311556 | C | C | T | Rv2052c | hypothetical protein |
| CP003248.2::3211587 | T | T | C | Rv2900c | formate dehydrogenase H FdhF |
| CP003248.2::3212635 | G | G | C | Rv2902c | ribonuclease HII protein RnhB |
| **Mixed Cluster 1** | H37Rv | 63 | 86 |  |  |
| CP003248.2::1489927 | A | A | G | Rv1325c | PE-PGRS family protein PE_PGRS24 |
| **Beijing Cluster 1** | H37Rv | 28 | 29 |  |  |
| CP003248.2::2268722 | A | A | C | Rv2024c  /Rv2023A | hypothetical proteins* |
| **Beijing Cluster 3** | H37Rv | 58 | 85 |  |  |
| CP003248.2::545710 | G | T | G | Rv0455c | hypothetical protein |
| CP003248.2::1720449 | G | G | A | Rv1525 | rhamnosyl transferase WbbL2 |
| CP003248.2::3840299 | C | C | A | Rv3224c | ATPase |
| **Beijing Cluster 5** | H37Rv | 74 | 77 |  |  |
| CP003248.2::3278307 | G | A | G | Rv2940c | mycocerosic acid synthase membrane-associated Mas |
| **H3 Cluster 1** | H37Rv | 76 | 78 |  |  |
| CP003248.2::900878 | C | C | T | Rv0806c | UDP-glucose-4-epimerase CpsY |
| CP003248.2::1422319 | C | C | T | Rv1273c | ABC transporter membrane protein |
| CP003248.2::4313334 | G | G | A | Rv3839 | hypothetical protein |
| **Manila-like Cluster 3** | H37Rv | 71 | 72 |  |  |
| CP003248.2::440562 | C | C | T | Rv0362 | Mg2+ transport transmembrane protein MgtE |

List of the SNPs that separate individual isolates within transmission clusters and the genes containing those SNPs. Isolates are listed by their University of Hawaii DNA Extraction Number. Gene designations and names were obtained from TubercuList. *Overlap of two hypothetical proteins in the reverse strand: 3' end of Rv2024c and 5' end of Rv2023A.
